# From niche topic to inclusion in the curriculum – design and evaluation of the elective course “climate change and health”

**DOI:** 10.3205/zma001613

**Published:** 2023-05-15

**Authors:** Dorothea Lemke, Svea Holtz, Meike Gerber, Olga Amberger, Dania Schütze, Beate Müller, Armin Wunder, Marischa Fast

**Affiliations:** 1 Goethe-University Frankfurt a.M., Institute of General Practice, Frankfurt a.M., Germany; 2 TU Dresden, Institut für Geschichte der Medizin, Dresden, Germany; 3 University of Cologne, Institute of General Practice, Cologne, Germany; 4 Medical University Graz, Institut für Allgemeinmedizin und Evidenzbasierte Versorgungsforschung, Graz, Austria; 5 Deutsche Allianz Klimawandel und Gesundheit (KLUG) e.V., Berlin, Germany

**Keywords:** climate change, health, medical education, evaluation, teaching

## Abstract

**Objective:**

At the Medical Faculty of the Goethe University Frankfurt am Main, the elective course “climate change and health” was offered to students in the clinical phase of their medical studies for the first time in the winter semester 2021/22 (any unfilled places were made available to interested students studying other subjects). Despite attracting considerable attention, this topic has not yet been incorporated into the curriculum of medical studies. Our aim was therefore to teach students about climate change and discuss its effects on human health. The students evaluated the elective in terms of various factors relating to knowledge, attitudes and behavior.

**Project description:**

The elective focused on the concept of Planetary Health, with an emphasis on the health consequences of climate change, as well as possibilities for action and adaptation in clinical and practical settings. The course took place in three live, online sessions (with inputs, discussion, case studies and work in small groups), as well as online preparation and a final written assignment for which students were asked to reflect on the subject.

The standardized teaching evaluation questionnaire (=didactic dimension) of Goethe University was used online to evaluate the elective, whereby the questionnaire was extended to include the measurement of changes in students’ agreement with items (dimensions) relating to knowledge, attitudes and behavior (personal behavior and behavior as physicians) before and after the course (pre/post comparison).

**Results:**

Students expressed high levels of satisfaction with the course content, the presentation of the course, and the organization of the elective. This was reflected in very good to good overall ratings. The pre/post comparisons further showed a significant, positive shift in agreement ratings in almost all dimensions. The majority of respondents also wanted the topic to be firmly embedded in the medical curriculum.

**Conclusion:**

The evaluation shows that with respect to the impact of climate change on human health, the elective course had a clear influence on the knowledge, attitudes, and behaviors of the students. In view of the relevance of the topic, it is therefore important that this subject is included in medical curricula in the future.

## Zusammenfassung

**Zielsetzung:**

Im Wintersemester 2021/22 wurde an der Medizinischen Fakultät der Goethe-Universität Frankfurt am Main zum ersten Mal das Wahlpflichtfach „Klimawandel und Gesundheit“ für Studierende der Humanmedizin im klinischen Abschnitt angeboten (Restplätze wurden an interessierte Studierende anderer Studiengänge vergeben). Ziel war eine Wissensvermittlung zu und Auseinandersetzung mit den Auswirkungen des Klimawandels auf die menschliche Gesundheit, da diese Thematik im Medizinstudium bislang nicht verankert ist, jedoch zunehmend an Brisanz gewinnt. Das Wahlpflichtfach wurde durch die Studierenden hinsichtlich verschiedener Wissens-, Einstellungs- und Verhaltensdimensionen evaluiert.

**Projektbeschreibung:**

Die inhaltlichen Schwerpunkte des Wahlpflichtfaches bildeten das Konzept Planetary Health, mit den Schwerpunkten der gesundheitlichen Folgen des Klimawandels sowie Handlungs- und Anpassungsmöglichkeiten in Klinik und Praxis. Durchgeführt wurde die Veranstaltung mittels drei Live-Online-Sitzungen (mit Inputs, Diskussion, Fall- und Kleingruppenarbeit) sowie Online-Vorbereitungsaufgaben und einer abschließenden schriftlichen Reflexion.

Für die Evaluation des Wahlpflichtfaches wurde die standardisierte Lehrevaluation (=didaktische Dimension) der Goethe-Universität als Online-Fragebogen verwendet. Dieser Fragenbogen wurde erweitert, um auch die Veränderungen der Zustimmung gegenüber Wissens-, Einstellungs- und Verhaltensdimensionen (persönliches und ärztliches Verhalten) vor und nach der Lehrveranstaltung (Prä/Post-Vergleich) zu messen.

**Ergebnisse:**

Die Studierenden äußerten sich sehr zufrieden bezüglich der präsentierten und vermittelten Inhalte, des Ablaufs und der Organisation des Wahlpflichtfaches, was sich in einer sehr guten bis guten Gesamtbewertung des Kurses abbildete. Außerdem wurden in den Prä/Post-Vergleichen in fast allen Dimensionen eine signifikante, positive Verschiebung der Zustimmungswerte gemessen. Die Mehrheit der Befragten wünschte sich zudem eine feste Verankerung der Thematik im Medizinstudium.

**Schlussfolgerung:**

Die Evaluation zeigt einen deutlichen Einfluss des durchgeführten Wahlpflichtfaches auf die Wissens-, Einstellungs- und Verhaltensdimensionen der Studierenden bezüglich der Auswirkungen des Klimawandels auf die menschliche Gesundheit. Daher wäre es mit Blick auf die Relevanz des Themas wichtig, diese Inhalte zukünftig in das medizinische Curriculum aufzunehmen.

## 1. Background

### 1.1. Introduction

Climate change is making itself felt in Germany through an increase in heat and extreme heat events (heat waves). The number of hot days (≥30°C) and tropical nights (≥20°C) has increased by an annual average of 11.4 days since 1950 (1950: 3 days, 2018: 20 days) [1], [2]. This trend is expected to continue. The steady increase in greenhouse gases in the atmosphere further raises thermal energy as a result of the greenhouse effect and is associated with a shift in the weather system towards extreme weather events. Such extreme weather events mainly include heat waves, droughts, heavy precipitation, and flooding [3]. This increase in extreme weather is associated with an increased health risk for everybody, but particularly for vulnerable population groups, such as the elderly, children or the chronically ill, who are particularly affected by the direct and indirect consequences of climate change [4], [5]. As a direct consequence of heat waves, for example, excess mortality as a result of cardiovascular disorders, kidney failure, respiratory diseases and strokes has been observed [5]. According to the Robert Koch Institute, mortality increases by between one and six percent per degree Celsius increase in temperature [5]. An increase in the burden of disease caused by infectious diseases is a further indirect health consequence. Among other things, this occurs because certain disease carriers penetrate into temperate zones as a result of global warming and, after being introduced, can spread to regions where they were not previously native. An example of this is the advance of the tiger mosquito, which is a carrier of the dengue and chikungunya viruses, into parts of Germany [6], [7]. Another indirect health consequence of global warming is an increase in allergies, which occurs because the increase in mean air temperature means pollen levels rise earlier in the year and last longer. Furthermore, neophytes that commonly cause allergies, such as mugwort ambrosia, may become increasingly widespread [7], [8], [9]. 

#### 1.2. Teaching on planetary health: The state of research

The concept of planetary health is based on the fundamental assumption that human health and the health of natural ecosystems are interdependent and cannot be considered in isolation from one another [10], [11]. There are widespread calls for information on planetary health to find its way into the training of health professionals, as they have a special responsibility for human health (ibid.). This demand is increasingly being taken seriously, often by medical students themselves, and especially with respect to the training of future physicians. It is important that physicians are well acquainted with the concept of planetary health, as well as the far-reaching consequences of climate change for human health, in order to be well prepared to practice medicine in a world in which climatic conditions are changing [12], [13], [14]. However, surveys among medical students revealed that although they consider the topic relevant to patient care, they lack knowledge about the overall way climate change is linked to human health and are thus in no position to talk about it with patients [15], [16], [17]. Although draft curricula already exist (some of which were developed by students themselves), the level of integration into medical curricula varies widely internationally [18]. For example, a review from the U.S. shows that while teaching on climate change and its health consequences is increasing, it is mainly limited to renowned research institutions, so access to these courses is unevenly distributed among students [19]. However, particularly in the US and UK, studies exist on various ways in which climate and health might be integrated into medical education [20], [21], [22]. One of the U.S. studies looked at a course on the environmental determinants of health, in which first-year medical students were asked to assess the health risks of a family they followed for an extended period of time [20]. An evaluation of this course offering showed that it improved medical students' self-assessed competence in discussing the health effects of climate change with patients. A similar course is available in South Africa, in which fourth-year medical students learn to assess environmental health risks in relation to the socioeconomic status of their patients [21]. In Germany, evidence-based research into how climate change and health, or planetary health courses, might be integrated into the medical curriculum, are lacking. In particular, knowledge about the importance of the topic and how it is related to their own role in health care is not widespread among students. For example, a study by Bugaj et al. indicates that medical students in their internship year certainly know about the health consequences of climate change, but they are unaware that they, as future physicians, may be considered professionally responsible for informing others about planetary health [15].

As the impact of the climate on human health is increasing, physicians need to be trained in both the diagnosis and treatment of diseases associated with climate and environmental changes. They should also be made aware of the influence of the health care system on climate change, especially considering that the health care sector is responsible for up to 5% of global greenhouse gas emissions [23]. Since the topic has not yet been incorporated into the medical curriculum at Goethe University Frankfurt, an elective course was made available to students in the clinical phase of their medical studies in the winter semester (WS) 2021/22 for the first time. After the registration deadline, any unfilled places were allocated to interested students from other fields of study, as long as some health connection could be established (e.g. sports science). The course was advertised using digital and analog postings on the medical campus. In the following, we present the concept of the elective and its evaluation. 

#### 1.3. Research questions 

The evaluation of the elective was based on responses to the following questions:

1. Did the elective help increase (subjectively assessed) knowledge about the links between climate change and health? (Knowledge dimension)
2. Do students feel the elective prepared them to assess and deal with health consequences of climate change? (Knowledge dimension)
3. What are students’ attitudes to climate change? (Attitude dimension)
4. In terms of their own self-efficacy, how effective do students consider their personal efforts to protect the climate to be, and did the elective influence their behavior? (personal behavior in the behavioral dimension).
5. How do students view their future role as physicians in the context of planetary health? (physician role behavioral dimension)
6. How did students assess the course from a didactical point of view? (Didactic evaluation dimension)

## 2. Project description

### 2.1. Description of the clinical elective “climate change and health”

The working group (WG) on Climate Change and Health at the Institute of General Practice decided to design an elective course for the clinical phase of medical studies at Goethe-University Frankfurt. Three members of the working group with differing areas of expertise (a physician, a qualified educationalist and a geoscientist) designed and implemented the elective. The design of the elective was based on a literature search to assess the current state of teaching on planetary health and on a discussion with experts from (amongst others) the working group on teaching at “Health for Future” and the working group on climate change and health at the German College for General Practitioners and Family Physicians (DEGAM).

The elective course entitled “climate change and health” was held in the winter semester of 2021/22 for the first time. It consisted of three live, online sessions lasting 3.5 teaching units (TU) each, which were preceded in each case by 1 TU on blended learning. The sessions took place in 14-day intervals (see attachment 1 ). Blended learning or integrated learning combines (in a didactically meaningful way) synchronous teaching formats with asynchronous online formats. By combining different formats and methods, the respective advantages of each method are enhanced [24]. 

All 20 places for students were filled. Interest in the topic also existed outside the medical department, so that in addition to 16 registrations from students in the clinical phase of their studies of human medicine, four students of other subjects also participated. However, two participants did not show up, so that a total of 18 students finally participated in the elective.

The elective consisted of three main topics:

1. Introduction to planetary health and geoscientific background information on climate change.
2. Adaptation: Links between the influence of climate and the environment on human health and its implications for patient care. The main focus thereby was on heat, with allergies and air pollution, infectious diseases, and mental health, as additional topics
3. Mitigation: The CO_2_ footprint of the healthcare system including ways of potentially reducing CO_2_ emissions in clinics and practices, the concept of climate-sensitive health counseling in patient consultations, as well as further possibilities to become active oneself.

The blended learning exercises that took place beforehand consisted of introductory online lectures and podcasts that prepared participants for the upcoming topic. As a result, it was possible to actively address the topic because enough time was available in the live, online sessions, not only for theoretical inputs but also for case studies and for work in small groups and general reflection. To ensure learning objectives had been reached, participants were required to write a reflection paper on a DIN A4 sheet of paper in response to the following questions: “What have I learned from the elective? What changes can I implement myself? What topics can I, as a physician/medical student, influence myself?” 

#### 2.2. Description of the questionnaire and data analysis

To evaluate the elective, the responsible interdisciplinary team extended the Goethe University’s teaching evaluation questionnaire and had it checked and tested for comprehensibility by independent scientists (not connected to the elective). The aim was not only to evaluate the didactic dimension of the course, but also the knowledge gain, attitudes to climate change and health effects, and behavioral attitudes, as well as the way participants viewed their personal behavior and their role as doctors. For this reason, the questionnaire contained an additional 37 closed questions (to be answered on a Likert scale) on the addressed dimensions: knowledge, attitudes, and behavior (divided into the dimensions of personal and physician behavior) in addition to a request for demographic information and questions on the didactics of the event. Questions in the knowledge, attitudes, and personal behavior dimensions were formulated as pre/post comparisons (e.g., “I had already dealt with the specific effects of climate change on health before the course” and “The course inspired me to take the specific effects of climate change on health more seriously”), so that a possible influence of the course on knowledge gain and any change in attitudes could be determined. The questionnaire was designed in EvaSys [https://evasys.de/en/] and a link to it was made available immediately after the final session. The data was then exported for analysis as a csv file. The original questionnaire can be found in attachment 2 (german version). An html version of this questionnaire was used in the evaluation.

As the number of cases was small, data analysis was mainly descriptive. Pre/post comparisons (before and after the course) were performed using the Wilcox test (one-sided and at a 5% significance level). For dependent samples, the Wilcox test is based on differences between the two groups. It considers not only the direction (positive/negative deviation, ranks), but also the magnitude of observed differences between the two groups (in this case, before and after the course). The effect size (r) was calculated in addition to statistical significance, as it provides an estimate of practical significance that helps in interpreting the size of an effect. Effect size was calculated for a single-group design with repeated measures. Values of less than 0.3 are interpreted as a small effect, between 0.3 and 0.5 as medium, and values greater than 0.5 as strong effects [https://rpkgs.datanovia.com/rstatix/reference/wilcox_effsize.html].

All analyses and graphical representations were performed using the R statistical program [https://www.r-project.org/].

#### 2.3. Ethics

Upon inquiry, the ethics committee said that as the survey was pseudonymized and conducted as part of the course evaluation, no extra committee vote was necessary.

## 3. Results of the evaluation

As 20 registrations were submitted, the course was fully booked. However, only 18 participants attended regularly, of whom 16 finally completed the evaluation. In terms of gender distribution, three participants were male (19%) and 13 were female. The median age of participants was 24 years and ranged from 21 to 34 years. Overall, 75% (n=12) of the respondents were enrolled and studying medicine in their 7^th^ to 11^th^ semester. Most of the medical students were in their 9^th^ semester (67%; n=8). 

### 3.1. Evaluation of the content and knowledge dimension

**Figure 1:**
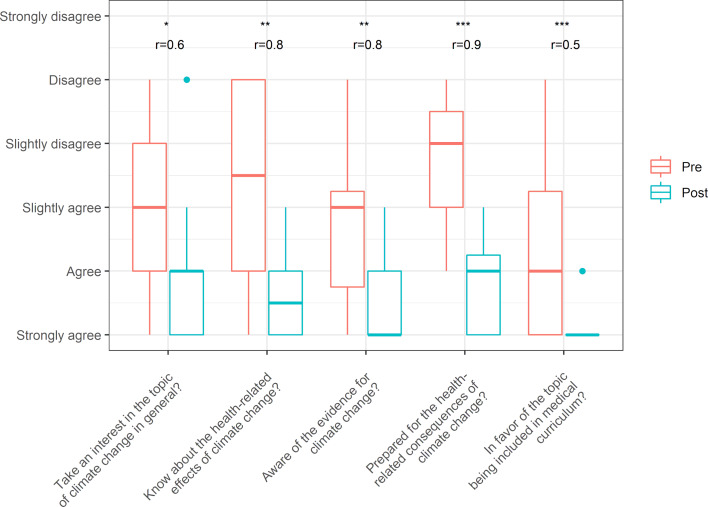
Distribution of responses before and after completion of the event in the knowledge dimension, including indication of significance (Wilcox Test, one-sided, alpha=0.05, *p<0.05, **p<0.01, ***p<0.001) and effect size (r)

In the content and knowledge dimension, respondents subjectively assessed the extent to which the course led to an increase in knowledge about climate change and health. The original questions can be found in the attached questionnaire in section 3 (see attachment 2 , german version). For clarity, the questions are abbreviated as topical items in figure 1 (Fig. 1) (x-axis). A significant difference in the assessment of knowledge before and after the event is shown for all the items (see figure 1 (Fig. 1)). However, the items concerning the estimation of the health consequences of climate change and the adoption of the topic in the medical curriculum showed a particularly significant increase in the assessment of knowledge before and after the event. In the estimation of health consequences, the effect size is especially high, which indicates that the event had a substantial influence. After completion of the elective, 81% of respondents gave their complete agreement that the subject should be firmly embedded in medical education. Before completion of the elective, the comparative figure was 38%. In discussions with participants, many had initially considered the elective to be a niche topic and were surprised in the end at the numerous specific effects of climate change on human health, as well as its effect on the work of physicians in the future and on the entire health system.

#### 3.2. Evaluation of the attitude dimension

**Figure 2:**
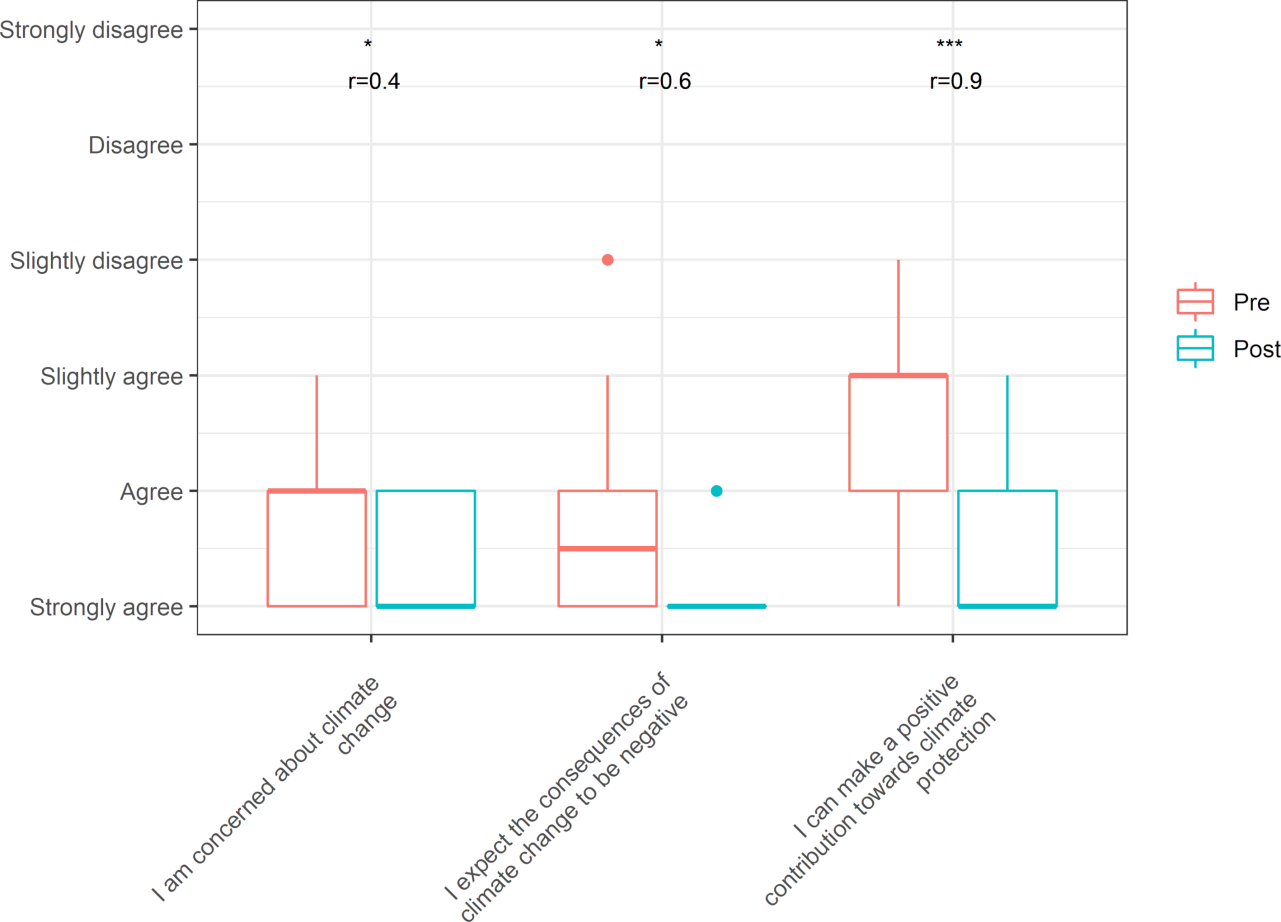
Distribution of responses before and after completion of the event in the attitude dimension, including significance level (Wilcox Test, one-sided, alpha=0.05, *p<0.05, **p<0.01, ***p<0.001) and indication of effect size (r)

The attitudinal dimension sought to discover whether changes in attitudes toward climate change and its health effects were reflected in a comparison between levels before and after the event (see figure 2 (Fig. 2)). The original questions can be found in section 4 of the questionnaire (see attachment 2 , german version). Agreement with concerns about climate change and its negative impact on life were stronger and more significant than before the event. The shift in agreement is particularly pronounced (and highly significant) for the statement that each individual can make a positive contribution to the environment, and the effect size is also very high.

#### 3.3. Evaluation of the behavioral dimension

**Figure 3:**
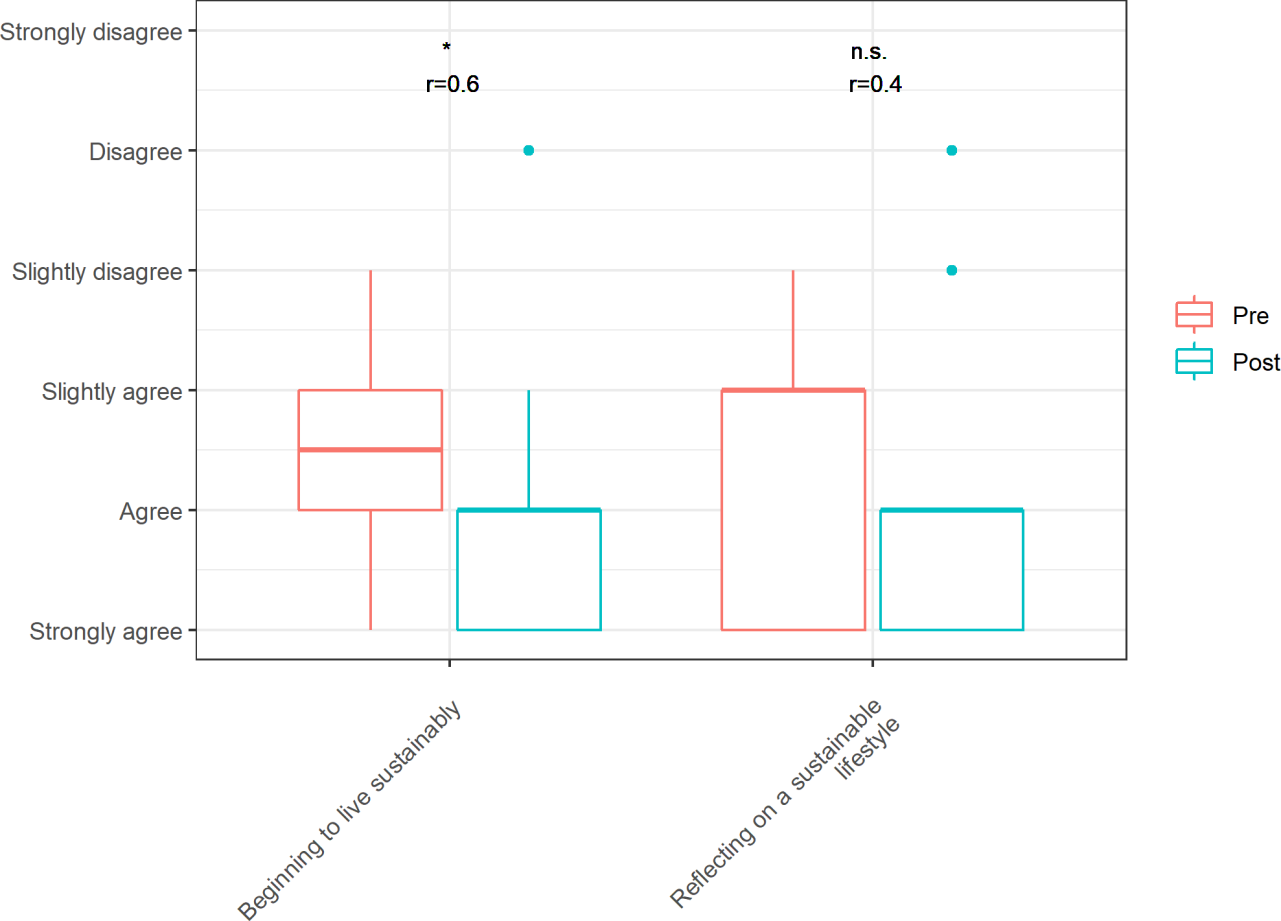
Distribution of responses before and after completion of the event in the behavioral dimension, including significance level (Wilcox Test, one-sided, alpha=0.05, *p<0.05, **p<0.01, ***p<0.001) and indication of effect size (r)

The behavioral dimension aimed to compare personal behavior with regard to sustainability before and after the course. The original questions can be found in section 6 of the questionnaire (see attachment 2 , german version). It could be shown that for the item “beginning to live sustainably” a significant shift in agreement with moderate effect sizes occurred. In contrast, the item “thinking about a sustainable way of life” showed no significant change in agreement (see figure 3 (Fig. 3)).

#### 3.4. Evaluation of role-related behavior as physicians

**Figure 4:**
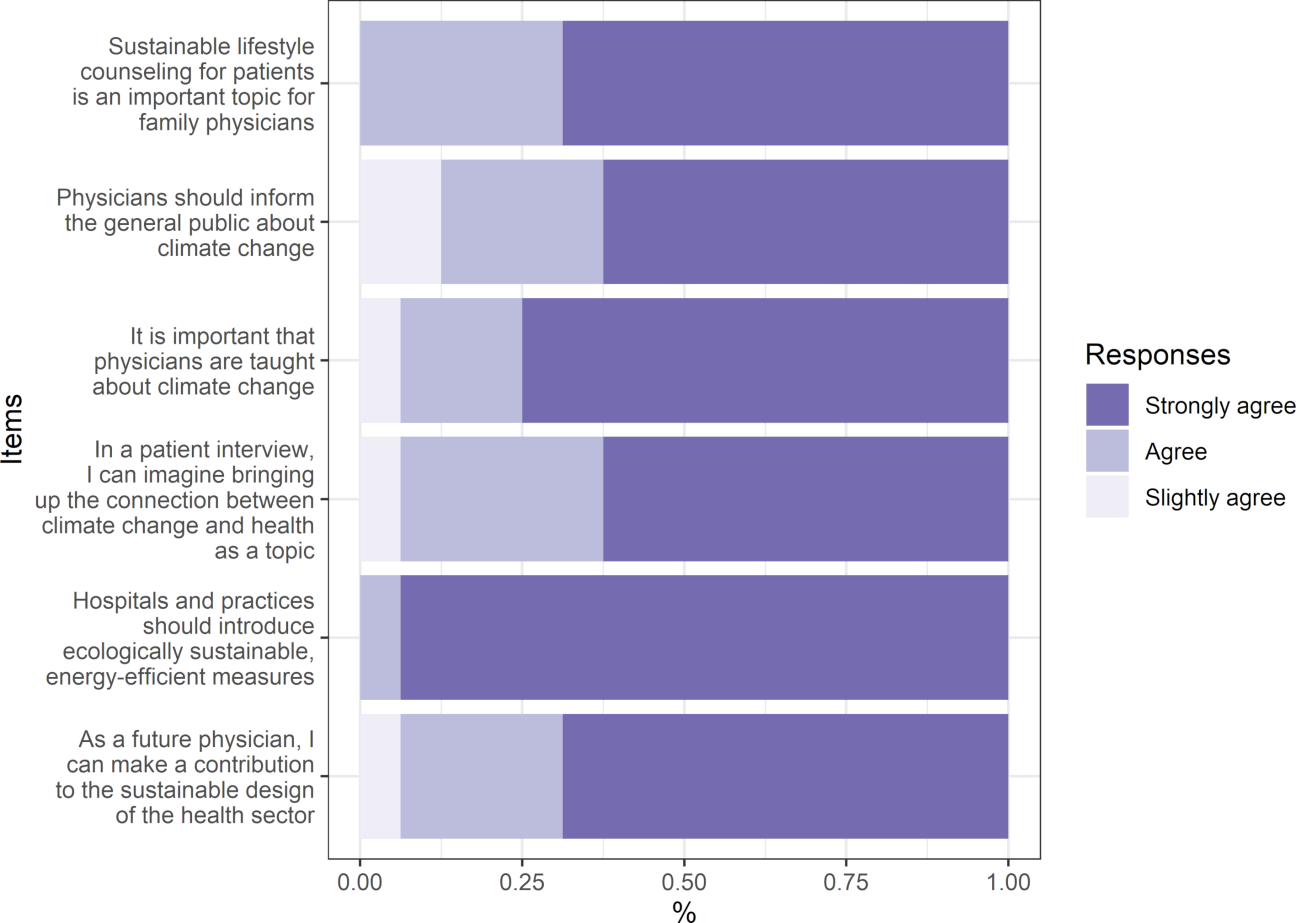
Influence of course content on (later) behavior as physicians

The dimension role-related behavior as physicians contains items designed to measure the probability that course content will find its way into the role-related behavior of the future physicians (see figure 4 (Fig. 4)). The four non-medical students were not excluded from participation in this evaluation because they were also enrolled in health-related courses and the items could also be considered relevant to their professions (e.g., sports science). The original questions can be found in section 5 of the questionnaire (see attachment 2 , german version). As the students could only anticipate their role-related behavior as physicians when the course took place, pre/post comparisons were not conducted. Particularly high levels of agreement were shown with the statement that hospitals and practices should be ecological, sustainable and energy efficient (94% completely agreed). Educating oneself about the health consequences of climate change was considered by 75% to be very important (complete agreement). In contrast, slightly fewer participants completely agreed (63%) that physicians should be responsible for educating the public on climate change. The same is true for educating patients on the relationship between climate change and health (63% completely agreed). Overall, around 69% agreed that future physicians can contribute to the sustainable design of the health sector, and that sustainable lifestyle counseling is an important topic for family physicians.

#### 3.5. Didactic evaluation

**Figure 5:**
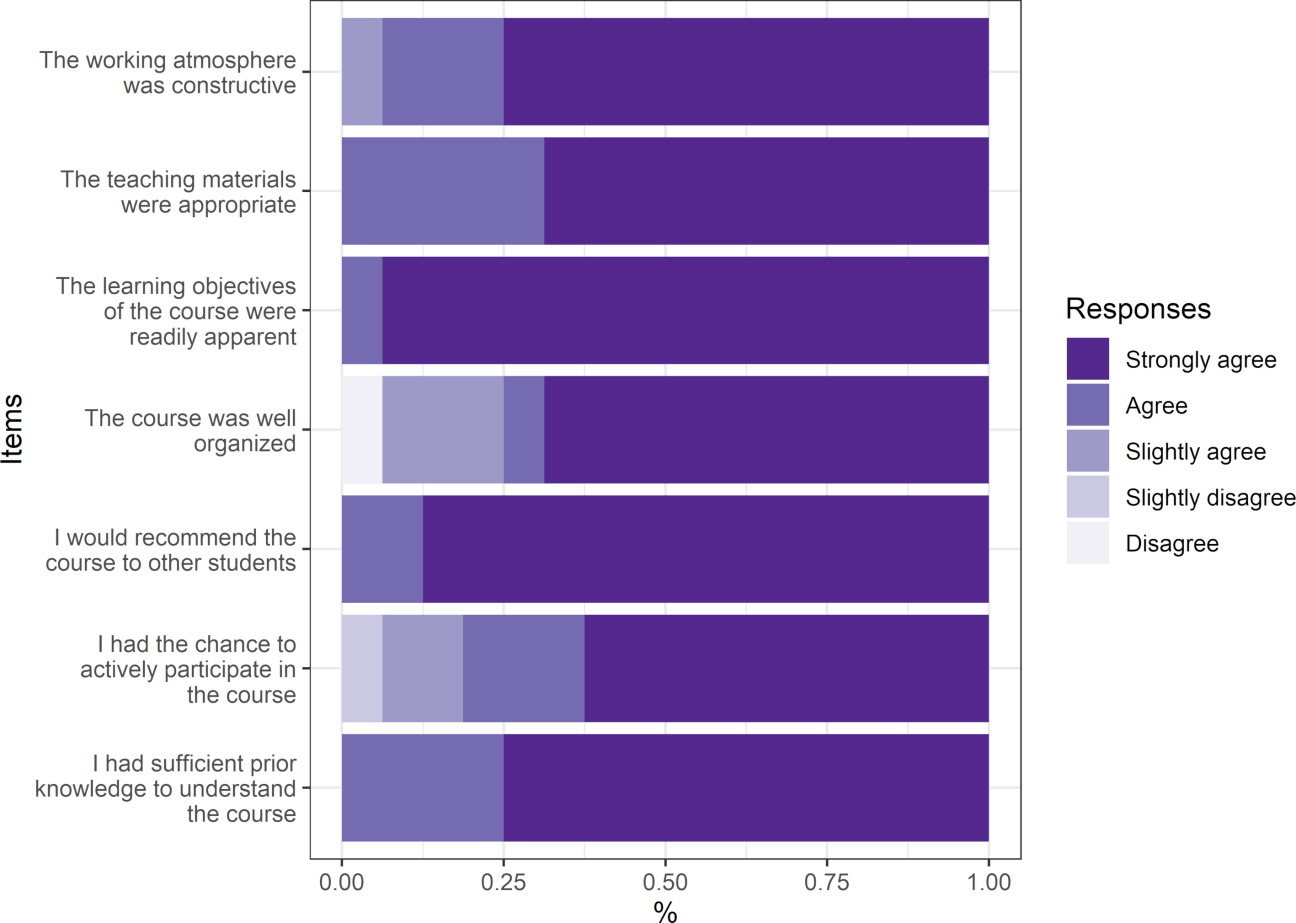
Didactic evaluation of the elective

The results of the didactic evaluation are shown in figure 5 (Fig. 5). The respondents were particularly positive about the possibility to participate actively in the course (94% completely agreed) and the constructive working atmosphere (88% completely agreed). The vast majority (>68%) “completely agreed” that their prior knowledge sufficed to follow the course, the teaching materials were satisfactory, and that the event was well organized. Around 75% of the respondents said they would recommend the event without reservation. The only question to be evaluated more diversely was whether the learning objectives were clearly recognizable to which 19% rather agreed or rather disagreed. The duration of the event was rated as “just right” by 70%, while 25% said it was too short and 5% too long. The amount of covered material was considered by 93% as “just right” and by 7% as too little. In all, 88% of respondents said they would be interested in participating in an advanced course on the same subject. In the overall evaluation, 56% gave the course a school grade of “1” and the rest a grade of “2”.

## 4. Discussion

The evaluation of the first clinical elective course on “climate change and health” offered by Goethe University Frankfurt am Main showed that participants were very satisfied with the course, that their subjectively assessed knowledge had increased significantly, and that their subjectively assessed attitudes and behavior towards the health consequences of climate change had changed significantly. After the course, the majority of respondents also wanted the topic to be firmly embedded in medical training. 

The results show that in terms of knowledge, attitudes, and behavior, the topic of climate change and its health consequences is not firmly rooted in either perceptions or medical education. This conclusion partly agrees with the results of the study by Bugaj et al. [15], who investigated the attitudes, as well as personal and professional role behavior, of final-year medical students with respect to climate change and health at Heidelberg University. For this purpose, a questionnaire was developed and factor analysis used to measure the attitudes of the final-year students in three dimensions: the expected consequences of climate change, personal, and professional (as physicians) responsibility. The study showed that agreement among the students was high for expected health risks and personal responsibility, but lower for professional responsibility. 

This is consistent with our findings that students, including final-year students, are more likely to make personal behavioral choices to mitigate the consequences of climate change but are less likely to see it as their professional role to educate the public about its (health) consequences. The fact that the students initially regarded the elective as a niche topic supports this view. This opinion and the knowledge deficits associated with the health effects of climate change and possibilities for physicians to do something about it show the importance of embedding the topic in the medical curriculum. Moreover, participants in the study can be assumed to be biased in the sense that students that were interested in the topic and knew more about it were more likely to have enrolled. It can therefore be assumed that the identified deficits will tend to be even more pronounced among medical students as a whole. The high approval ratings of the suggestion to include the topic in the medical curriculum indicates that participants felt their knowledge to date had been insufficient. 

Initiatives to include the topic of planetary health in the National Catalogue of Learning Objectives in Medicine (NLKM), and therefore to include it as a subject for examination, further reflect the necessity and, above all, the urgency of ensuring that the topic is considered mandatory in medical training [25]. 

The evaluation by Gomez et al. [26] of the introduction of a course on the impact of climate change on human health at the Stanford School of Medicine also showed that after the course, the idea to make “climate change” a subject to be included in medical training met with significant approval. There was also a significant shift in awareness of the health impact of climate change after completion of the course, which was also evident in the present evaluation. Further comparisons of our results with the literature are difficult because little research has been conducted on the topic in Germany and, internationally, the available courses offered by medical departments on the subject, and the items/questions that are covered, vary widely. 

The limitations of the teaching evaluation presented here are primarily that the sample is small and presumably self-selected, since the course was not compulsory and participants were presumably already interested in the topic. In addition, participants were only surveyed at the end of the course. The pre/post comparisons may therefore be biased, since the survey was conducted retrospectively with respect to the pre time point. Additional limitations are that data is not yet available for group comparisons, and that the results may reflect self-reporting bias, i.e., a shift in responses toward social desirability. Longitudinal studies will be necessary to investigate changes in knowledge and behavior and possibly also to measure any specific contribution to health care.

## 5. Conclusion

Overall, the evaluation shows that awareness of the relationship between climate change and human health is clearly inadequate, and that courses on the subject can have an important influence on knowledge levels, attitudes and behaviors. Based on the current state of research and our own evaluation, as well as the desire of the students to firmly embed the topic in medical studies and to learn more about it, we would recommend that courses on climate change and health are included in medical curricula. 

## Acknowledgements

The authors wish to thank Phillip Elliott for translating the manuscript. 

## Competing interests

The authors declare that they have no competing interests. 

## Supplementary Material

Overview of the content of the elective and the course procedure

Questionnaire
