# Supplementary material for: From niche topic to inclusion in the curriculum – design and evaluation of the elective course “climate change and health”
Source: GMS J Med Educ. 2023 May 15;40(3):Doc31. doi: 10.3205/zma001613 (PMC10291346; doi:10.3205/zma001613)
Supplement: Overview of the content of the elective and the course procedure [file JME-40-31-s-001.pdf]

Attachment 1: Overview of the content of the elective and the course procedure

	Content	Sequence of events and didactic methods	Blended Learning tasks (1 teaching unit each)
**First session** (3.5 teaching units), live and online via Zoom	Introduce yourself and explain how you come to be involved in the topic. Clarification of expectations; Introduction to the topic of planetary health; geoscientific background information on climate change	Short round of introductions and clarification of the process and learning objectives (plenary). Watch the introductory lecture of the Planetary Health Academy with the speakers Prof. Harald Lesch, Dr. Martin Herrmann and Prof. Sabine Gabrysch. Small moderated group discussion of the questions: "With regard to the lecture and the talk by Sabine Gabrysch: What is going through my mind? What questions and open issues do I have, what do I want to find out more about/address?" Information subsequently collected on the whiteboard. Input lecture on "Climatology and evidence of climate change". Quick feedback to the question "What have I learned today?"	Preparation: Video lecture: "How do things stand for the earth and human life? Planetary health – a comprehensive health concept" by Sabine Gabrysch am 21.01.2021
**Second session** (3.5 teaching units), live and online via Zoom	Adaptation: Explain the links between the impact of the climate and environment on human health and its implications for patient care, with a focus on heat. Additional topics include allergies and air pollution, infectious diseases, and mental health.	Welcome participants and provide an overview of the topics and objectives. Review of self-study with presentation of results on mentimeter. Input lecture "Health consequences of climate change, focusing on heat". Group work (4 small groups) on the topic of heat based on a case study and compilation of results. Break and activation. Input lecture "Allergies and air pollution, infectious diseases, air pollution, mental health" followed by discussion and quiz on the topic. Quick feedback to the question "What have I learned today?"	Preparation: Amboss podcast: "Climate change and health – an appeal (1)" with Dr. Eckart von Hirschhausen With the task of selecting three particularly important aspects from the podcast and entering them into *mentimeter*.

Attachment 1 to Lemke D, Holtz S, Gerber M, Amberger O, Schütze D, Müller B, Wunder A, Fast M. *From a niche topic to inclusion in the curriculum – design and evaluation oft he elective course "climate change and health"*. GMS J Med Educ. 2023;40(3):Doc31. DOI: 10.3205/zma001613

	Content	Sequence of events and didactic methods	Blended Learning tasks (1 teaching unit each)
**Third session** (3.5 teaching units), live and online via Zoom	Mitigation: The CO2 footprint of the health system including measures to potentially reduce CO2 in the clinic and practice. The concept of climate-sensitive health counseling in patient consultations. Other ways of being active oneself. End of the elective	Welcome participants and provide an overview of the topics and objectives. During the discussion, collect responses to the question, "What do I wish for today, what would be a *fitting* conclusion to the elective for me?" Input lecture "Mitigation – CO2 footprint of the health system – sustainable practices and hospitals" (in addition to the workshop instructors, a family physician in private practice was invited to report on his sustainable practice). Individual work and moderated group word on the topic, "Choose an aspect aimed at making your clinic or practice more sustainable and think about how you would convince your boss of your idea in 30 seconds.", followed by demonstrating your persuasive speech to the small group, and receiving feedback. Break and activation. Input lecture "Climate-sensitive health counseling". Casework on climate-sensitive health counseling in small groups with subsequent reports in the plenum. Mini input: Why be active with a presentation by the Health for Future group Frankfurt a.M. (by an activist from the group that has been invited); Final reflection in three small groups on the question, "What have I learned from the elective course?" Conclusion and leave-taking.	Preparation: Video lecture "Climate crisis – a medical emergency" by Dr. Martin Herrmann on 04.11.2019<br><br>Test whether learning objectives have been reached: Written reflection on one page of DIN A4 paper on the questions, "What have I learned from the elective course? What can I put into practice? As a future physician, what topics can I influence?
**Test of attainment of learning objectives**	Reflect on the questions, "What have I learned from the elective course? What can I put into practice? As a future physician, what topics can I influence?“	Written reflection on one page of DIN A4 paper.	
