# Supplementary material for: From niche topic to inclusion in the curriculum – design and evaluation of the elective course “climate change and health”
Source: GMS J Med Educ. 2023 May 15;40(3):Doc31. doi: 10.3205/zma001613 (PMC10291346; doi:10.3205/zma001613)
Supplement: Questionnaire [file JME-40-31-s-002.pdf]

Attachment 2: Questionnaire

Attachment 2 to Lemke D, Holtz S, Gerber M, Amberger O, Schütze D, Müller B, Wunder A, Fast M. *From a niche topic to inclusion in the curriculum – design and evaluation oft he elective course "climate change and health"*. GMS J Med Educ. 2023;40(3):Doc32. DOI: 10.3205/zma001614

MUSTER

evasys	Evaluation des Wahlfaches [Copy] [Copy] [Copy]	evasys

Institut für Allgemeinmedizin

Klimawandel und Gesundheit

Bitte so markieren: ☐ ☒ ☐ ☐ ☐ Bitte verwenden Sie einen Kugelschreiber oder nicht zu starken Filzstift. Dieser Fragebogen wird maschinell erfasst.

Korrektur: ☐ ■ ☐ ☒ ☐ Bitte beachten Sie im Interesse einer optimalen Datenerfassung die links gegebenen Hinweise beim Ausfüllen.

Liebe Studierende,
bitte füllen Sie diesen Fragebogen so vollständig aus wie möglich. Wir sind an Ihrer persönlichen Wahrnehmung interessiert und möchten anhand dieser stets unsere Lehre verbessern. Alle Angaben sind freiwillig. Vielen Dank für Ihre Unterstützung!

1. Angaben zur Person

1.1 **Erster** Buchstabe des **Vornamens Ihrer Mutter**

a

1.2 **Erster** Buchstabe des Studienortes, an dem Sie Medizin / Pharmazie studierten

b

1.3 Geburts**tag** Ihrer Mutter (**TT**/MM/JJJJ)

cc

1.4 **Erster** Buchstabe Ihres **Geburtsortes**

d

1.5 Geschlecht ☐ Weiblich ☐ Männlich ☐ Divers

1.6 Geburtsjahr

1.7 Studiengang ☐ Medizin ☐ Sonstiges

1.8 Semester

1.9 Thema der Veranstaltung:

1.10 Datum

Tag Monat Jahr

MUSTER

2. Bewertung des Kurses

Bitte bewerten Sie den Kurs, indem Sie jeweils ein Feld pro Aussage ankreuzen

		stimme völlig zu	stimme zu	stimme eher zu	stimme eher nicht zu	stimme nicht zu	
2.1	Die Lernziele der Veranstaltung waren klar erkennbar	☐	☐	☐	☐	☐	☐
2.2	Ich hatte die Möglichkeit, mich aktiv an der Veranstaltung zu beteiligen	☐	☐	☐	☐	☐	☐
2.3	Die Arbeitsatmosphäre war konstruktiv	☐	☐	☐	☐	☐	☐
2.4	Die verwendeten Unterrichtsmaterialien waren angemessen	☐	☐	☐	☐	☐	☐
2.5	Ich hatte ausreichende Vorkenntnisse, um der Veranstaltung zu folgen	☐	☐	☐	☐	☐	☐
2.6	Die Veranstaltung war gut organisiert	☐	☐	☐	☐	☐	☐
2.7	Ich würde diese Veranstaltung anderen Studierenden weiterempfehlen	☐	☐	☐	☐	☐	☐

2.8	Der Zeitrahmen der Veranstaltung war	☐ zu kurz	☐ genau richtig	☐ zu lang
2.9	Die Stoffmenge der Veranstaltung war	☐ zu wenig	☐ genau richtig	☐ zu viel
2.10	Ich habe Interesse an einem weiterführenden Aufbaukurs zu diesem Thema	☐ ja	☐ nein	

2.11 **Gesamtnote** für diese Veranstaltung (in Schulnoten 1 = sehr gut bis 6 = ungenügend):

2.12 Folgende Themen würde ich zusätzlich in die Veranstaltung mit aufnehmen oder ausführlicher behandeln:

2.13 Ich habe folgendes Feedback für die Unterrichtsmaterialien und -methoden:

3. Inhalte/Wissen

3. Inhalte/Wissen [Fortsetzung]

		stimme völlig zu	stimme zu	stimme eher zu	stimme eher nicht zu	stimme nicht zu	stimme überhaupt nicht zu
3.1	Vor dem Kurs habe ich mich bereits mit dem Thema Klimawandel allgemein beschäftigt	☐	☐	☐	☐	☐	☐
3.2	Nach Abschluss des Kurses werde ich mich mehr mit dem Thema Klimawandel zu beschäftigen	☐	☐	☐	☐	☐	☐
3.3	Vor dem Kurs habe ich mich bereits mit den Auswirkungen des Klimawandels auf die Gesundheit beschäftigt.	☐	☐	☐	☐	☐	☐
3.4	Nach Abschluss des Kurses werde ich mich stärker mit den gesundheitlichen Auswirkungen des Klimawandels zu beschäftigen	☐	☐	☐	☐	☐	☐
3.5	Vor dem Kurs kannte ich bereits die Beweise/Evidenz für den Klimawandel	☐	☐	☐	☐	☐	☐
3.6	Nach Abschluss des Kurses kenne ich die Beweise/ Evidenz für den Klimawandel.	☐	☐	☐	☐	☐	☐
3.7	Vor dem Kurs fühlte ich mich bereits gut vorbereitet, die gesundheitlichen Folgen des Klimawandels abzuschätzen und damit umzugehen	☐	☐	☐	☐	☐	☐
3.8	Nach Abschluss des Kurses fühle ich mich gut darauf vorbereitet, die gesundheitlichen Folgen des Klimawandels abzuschätzen und damit umzugehen	☐	☐	☐	☐	☐	☐
3.9	Vor dem Kurs war ich schon der Meinung, dass Klimawandel und Gesundheit fester Bestandteil in der medizinischen Ausbildung/Curriculum werden sollte.	☐	☐	☐	☐	☐	☐
3.10	Nach Abschluss des Kurses bin ich der Meinung, dass Klimawandel und Gesundheit fester Bestandteil in der medizinischen Ausbildung/Curriculum werden sollte.	☐	☐	☐	☐	☐	☐

4. Einstellungen (allgemein)

		stimme völlig zu	stimme zu	stimme eher zu	stimme eher nicht zu	stimme nicht zu	stimme überhaupt nicht zu
4.1	Vor dem Kurs war ich schon besorgt über den Klimawandel und dessen Auswirkungen.	☐	☐	☐	☐	☐	☐
4.2	Nach Abschluss des Kurses bin ich besorgt über den Klimawandel und dessen Auswirkungen.	☐	☐	☐	☐	☐	☐
4.3	Der Klimawandel wirkt sich heute schon negativ auf unser Leben aus	☐	☐	☐	☐	☐	☐

4. Einstellungen (allgemein) [Fortsetzung]

4.4	Der Klimawandel wird sich auf zukünftige Generationen negativ auswirken.	☐	☐	☐	☐	☐	☐
4.5	Vor dem Kurs war mir schon bewusst, dass ich einen positiven Beitrag für die Umwelt leisten kann.	☐	☐	☐	☐	☐	☐
4.6	Nach Abschluss des Kurses ist mir bewusst, dass ich einen positiven Beitrag für die Umwelt leisten kann.	☐	☐	☐	☐	☐	☐

5. Ärztliches Verhalten

		stimme völlig zu	stimme zu	stimme eher zu	stimme eher nicht zu	stimme nicht zu	stimme überhaupt nicht zu
5.1	Es ist wichtig, dass Ärzt:innen über den Klimawandel aufgeklärt werden	☐	☐	☐	☐	☐	☐
5.2	Ärzt:innen sollten die Öffentlichkeit über den Klimawandel informieren	☐	☐	☐	☐	☐	☐
5.3	Krankenhäuser und Praxen sollten ökologisch nachhaltige, energieeffiziente Praktiken einführen	☐	☐	☐	☐	☐	☐
5.4	Nachhaltige Lebenstilberatung bei Patient:innen ist ein wichtiges hausärztliches Thema	☐	☐	☐	☐	☐	☐
5.5	Als zukünftige:r Ärzt:in kann ich einen Beitrag zur nachhaltigen Gestaltung des Gesundheitssektor leisten	☐	☐	☐	☐	☐	☐
5.6	Ich kann mir vorstellen die Zusammenhänge zwischen Klimawandel und Gesundheit im Patient: innen-Gespräch zu thematisieren.	☐	☐	☐	☐	☐	☐

6. Persönliches Verhalten

		stimme völlig zu	stimme zu	stimme eher zu	stimme eher nicht zu	stimme nicht zu	stimme überhaupt nicht zu
6.1	Vor dem Kurs habe ich bereits über einen nachhaltigeren Lebensstil nachgedacht.	☐	☐	☐	☐	☐	☐
6.2	Der Kurs hat mich angeregt über einen nachhaltigeren Lebensstil nachzudenken.	☐	☐	☐	☐	☐	☐
6.3	Vor dem Kurs habe ich bereits angefangen mein Leben nachhaltiger zu gestalten (Ernährung, Mobilität, Müll).	☐	☐	☐	☐	☐	☐
6.4	Der Kurs hat mich angeregt mein Leben nachhaltiger zu gestalten (Ernährung, Mobilität, Müll)	☐	☐	☐	☐	☐	☐
6.5	Ich glaube, die im Kurs erlernten Inhalte in meinem Beruf umsetzen zu können.	☐	☐	☐	☐	☐	☐
6.6	Der Kurs hat mich dazu angeregt, mich zukünftig aktiv für das Thema einzusetzen.	☐	☐	☐	☐	☐	☐

MUSTER

6. Persönliches Verhalten [Fortsetzung]

6.7 Für diese Themen würde ich mich zukünftig einsetzen:

7.

7.1 **Grundsätzliches Feedback zur Veranstaltung**

Vielen Dank für Ihre Mithilfe und viel Erfolg für Ihr Studium!

MUSTER

SAMPLE
Evaluation of elective course
Institute of General Practice
Climate change and health
Please mark in this way: Please use a ballpoint pen or a felt pen that is not too thick.
The questionnaire will be digitalized and recorded.

Correction: To ensure the data is recorded as accurately as possible, please follow the instructions on filling in the questionnaire on the left.
Dear students,
Please complete the questionnaire as fully as possible. We are interested in your thoughts and would like use them to help improve our teaching. All information is provided voluntarily. Thank you for your support!

1. Personal details
1.1 First letter of your mother's first name
a
1.2 First letter of the place where you are studying medicine/pharmacy
b
1.3 Birthday of your mother (TT/MM/JJJJ)
cc
1.4 First letter of the place you were born
d
1.5 Sex female male other
1.6 Year of birth
1.7 Course of study medicine other
1.8 Semester
1.9 Course topic:
1.10 Date
Day Month Year
Institute of General Practice
Climate change and health
Evaluation of elective

2. Evaluation of course
Please evaluate the course by putting a cross in one of the boxes for each statement
Strongly agree
Agree
Slightly agree
Slightly disagree
Disagree
2.1 The learning objectives of the course were readily apparent
2.2 I had the chance to actively participate in the course
2.3 The working atmosphere was constructive
2.4 The teaching materials used in the course were appropriate
2.5 I had sufficient prior knowledge to understand the course
2.6 The course was well organized
2.7 I would recommend the course to other students
2.8 The duration of the course was too short absolutely right too long

2.9 The quantity of material covered in the course was too little absolutely right too much
2.10 I would be interested in an advanced course on the same subject yes no
2.11 Overall grade for the course (using school grading system 1 = very good to 6 = unsatisfactory):
2.12 I would include the following topics in the course or deal with them in more detail:
2.13 I have the following feedback concerning the teaching materials and methods

3. Content/Knowledge
Evaluation of elective
3. Content/Knowledge [cont.]
Strongly agree
Agree
Slightly agree
Slightly disagree
Disagree
Strongly disagree
3.1 Before the course, I had already explored the topic of climate change in a general way
3.2 Following the course, I will study the topic of climate change in more depth
3.3 Before the course, I had already studied the effects of climate change on health.
3.4 Following the course, I will study the effects of climate change on health in more depth
3.5 Before the course, I was already aware of the proof/evidence for climate change
3.6 After completing the course, I am aware of the proof/evidence for climate change.
3.7 Before the course, I felt well prepared to assess and face the health consequences of climate change
3.8 Following the course, I feel well prepared to assess and face the health consequences of climate change
3.9 Before the course, I was already of the opinion that climate change and health should be firmly embedded into the curriculum of medical studies.
3.10 Following the course, I am of the opinion that climate change and health should be firmly embedded into the curriculum of medical studies.
4. Attitudes (general)
Strongly agree
Agree
Slightly agree
Slightly disagree
Disagree
Strongly disagree
4.1 Before the course I was already concerned about climate change and its consequences.
4.2 Following the course, I am concerned about climate change and its consequences.
4.3 Climate change is already having a negative impact on our lives.
Evaluation of the elective
4. Attitudes (general) [cont.]
4.4 Climate change will have negative consequences for future generations.

4.5 Before the course, I was already aware that I could make a positive contribution for the environment.
4.6 Following the course, I am aware that I can make a positive contribution for the environment.
5. Physician behavior
Strongly agree
Agree
Slightly agree
Slightly disagree
Disagree
Strongly disagree
5.1 It is important that physicians are taught about climate change.
5.2 Physicians should tell the general public about climate change.
5.3 Hospitals and practices should introduce ecologically sustainably, energy-efficient measures.
5.4 Sustainable lifestyle counseling for patients is an important topic in family medicine.
5.5 As a future physician, I can make a contribution towards the sustainable design of the health system.
5.6 I can imagine bringing up the association between climate change and health and discussing it in patient interviews.
6. Personal behavior
Strongly agree
Agree
Slightly agree
Slightly disagree
Disagree
Strongly disagree
6.1 Before the course, I had already considered adopting a more sustainable lifestyle.
6.2 The course encouraged me to consider adopting a more sustainable lifestyle.
6.3 Before the course, I had already begun to live more sustainably (nutrition, mobility, trash).
6.4 The course encouraged me to live more sustainably (nutrition, mobility, trash).
6.5 I believe I will be able to use what I learned in the course in my profession.
6.6 The course encouraged me to become more actively involved in the topic.

6. Personal behavior [cont.]
6.7 I will involve myself more actively in these topics in the future:
7.
7.1 Basic feedback on the course
Thank you for your support and we wish you every success with your studies!
